# Reply

**DOI:** 10.1016/j.jvsv.2026.102472

**Published:** 2026-06-22

**Authors:** Yun Sun Lim, Prajwala S. Prakash, Marjorie T.Q. Hoang, Ruijie Li, Li Zhang, Enming Yong

**Affiliations:** Yong Loo Lin School of Medicine, National University of Singapore, Singapore; Vascular Surgery Service, Department of General Surgery, Tan Tock Seng Hospital, Singapore; Department of General Surgery, Sengkang General Hospital, Singapore; Health Services and Outcomes Research, National Health Care Group, Singapore; Vascular Surgery Service, Department of General Surgery, Tan Tock Seng Hospital, Singapore

We thank Mahida et al for their perceptive letter, and sincerely appreciate their recognition of our recent work on optimizing long-term outcomes following iliac vein stenting for symptomatic iliofemoral venous obstruction.^1^ We agree that the potential contributions of inflow disease and anticoagulation noncompliance to early stent failure are key mechanistic considerations, as they aptly highlighted.

In response to Mahida et al’s comment regarding the risk factors for stent failure in our multivariate analysis, which included post-thrombotic syndrome (PTS) and non-thrombotic iliac vein lesions, we wish to clarify that the observed hazard ratio (HR) for PTS should be interpreted as very weak evidence for causality. PTS likely serves as a proxy for more advanced and severe inflow disease, which predisposes patients to stent occlusions. This aligns with Jalaie’s finding that primary patency loss correlates with the anatomical classification of chronic venous obstruction (CVO).^2^ We have categorized stent occlusions by Jalaie’s CVO classification of the iliofemoral tract (Table). However, the lack of patients with CVO types 4 and 5 meant that no estimates could be made about their relative risk or HRs. The presence of a single patient in CVO type 3 with no occluded stent indicated that the relative risk/HR will not be meaningful, and no confidence interval can be calculated with a single patient. We considered grouping Jalaie types 2 and 3 and comparing them against type 1 as well, but this would have essentially replicated our PTS vs non-thrombotic iliac vein lesions analysis. We look forward to future prospective studies with larger samples in validating this anatomical CVO classification to establish association between inflow severity and stent occlusion, which will allow better predictions of stent outcomes.

**Table:** Distribution of stent occlusions across Jalaie chronic venous obstruction (*CVO*) types

Jalaie CVO classification	Patients	Occluded stents, frequency
Type 1	81 (NIVL)	5
Type 2	8 (PTS)	2
Type 3	1 (PTS)	0
Type 4	0	0
Type 5	0	0

*NIVL,* Non-thrombotic iliac vein lesion; *PTS,* post-thrombotic syndrome.

Incomplete swelling relief despite high venous stent patency rates may be attributed to concomitant comorbidities such as renal or cardiac disease.^3^ Furthermore, up to 30% of patients with chronic venous disease also have coexisting lymphatic dysfunction. Both points are emphasized by Raju et al, who showed that complete swelling relief post iliac vein stenting is low to moderate at 16% and 44% (*P* < .001); partial improvement (≥1 grade of swelling) of 45% and 66% (*P* < .01) in the abnormal and normal lymphangiographic groups, respectively,^4^ similar to our series’ reported rates of sustained swelling relief at 53.1%. Nonetheless, we agree that future analyses with post-treatment reflux mapping or validated quality of life instruments could distinguish anatomical success from true clinical resolution.

Finally, we acknowledge that the classification for stent occlusions was based on retrospective documentation of anticoagulation factors. Routine drug level monitoring is not currently standard of care for anticoagulation, particularly with direct oral anticoagulants, due to their predictable pharmacokinetics. Nevertheless, measuring anticoagulant activity^5^ in specific clinical cases such as venous stent occlusion or in venous stenting patients with extremes of body weight, renal impairment, or bariatric surgery may be an area of future venous surgery research.

## Funding

None.

## Disclosures

None.
